# CpGs Induce Differentiation of Atlantic Salmon Mononuclear Phagocytes Into Cells With Dendritic Morphology and a Proinflammatory Transcriptional Profile but an Exhausted Allostimulatory Activity

**DOI:** 10.3389/fimmu.2019.00378

**Published:** 2019-03-13

**Authors:** Dimitar B. Iliev, Leidy Lagos, Hanna L. Thim, Sven M. Jørgensen, Aleksei Krasnov, Jorunn B. Jørgensen

**Affiliations:** ^1^The Norwegian College of Fishery Science, UiT The Arctic University of Norway, Tromsø, Norway; ^2^Department of Gene Regulation, Institute of Molecular Biology ‘Roumen Tsanev’, Bulgarian Academy of Sciences, Sofia, Bulgaria; ^3^Nofima Marin AS, Oslo, Norway

**Keywords:** mononuclear phagocytes, antigen-presenting cells, dendritic cells, CpG oligodeoxynucleotides, Atlantic salmon, teleosts

## Abstract

Due to their ability to present foreign antigens and prime naïve T cells, macrophages, and dendritic cells (DCs) are referred to as professional antigen-presenting cells (APCs). Although activated macrophages may function as APCs, these cells are particularly effective at directly engaging pathogens through phagocytosis, and production of antimicrobial compounds. On the other hand, DCs possess superb antigen-presenting and costimulatory capacity and they are essential for commencement and regulation of adaptive immune responses. In *in vitro* models, development of mature mammalian DCs from monocytes requires sequential exposure to growth factors (including GM-CSF and IL-4) and proinflammatory stimuli such as toll-like receptor (TLR) ligands. Currently, except for IL-4/13, neither orthologs nor functional analogs of the growth factors which are essential for the differentiation of mammalian DCs (including GM-CSF and FLT3) have been identified in teleosts and data about differentiation of piscine APCs is scant. In the present study, primary salmon mononuclear phagocytes (MPs) stimulated *in vitro* for 5–7 days with a B-class CpG oligodeoxynucleotides (ODN 2006PS) underwent morphological differentiation and developed “dendritic” morphology, characterized by long, branching pseudopodia. Transcriptional profiling showed that these cells expressed high levels of proinflammatory mediators characteristic for M1 polarized MPs. However, the cells treated with CpGs for 7 days downregulated their surface MHCII molecules as well as their capacity to endocytose ovalbumin and exhibited attenuated allostimulatory activity. This concurred with transcriptional downregulation of costimulatory *CD80/86* and upregulation of inhibitory *CD274 (B7-H1)* genes. Despite their exhausted allostimulatory activity, these cells were still responsive to re-stimulation with gardiquimod (a TLR7/8 ligand) and further upregulated a wide array of immune genes including proinflammatory mediators such as *intereukin-1 beta* and *tumor necrosis factor*. Overall, the presented data highlight the disparate effects TLR ligands may have on the proinflammatory status of APCs, on one side, and their antigen-presenting/costimulatory functions, on the other. These findings also indicate that despite the poor phylogenetic conservation of the growth factors involved in the differentiation of DCs, some of the processes that orchestrate the development and the differentiation of professional APCs are conserved between teleosts in mammals.

## Introduction

The mononuclear phagocyte (MP) system comprises circulating monocytes, tissue resident macrophages, and monocyte-derived DCs. The most important unifying properties of MPs are their common myeloid origin and their potential to serve as professional APCs. These professional APCs are innate immune cells; however they are very important for the commencement of the adaptive immune response (1, 2). More specifically, they are indispensable for the activation of naïve T cells by protein antigens and are essential for the maintenance of tolerance to self-antigens. The MP system is phylogenetically conserved and cells resembling mammalian monocytes, macrophages, and DCs have been identified across different vertebrate classes (3–5).

Although macrophages are mainly engaged in direct elimination of pathogens through endocytosis and production of antibacterial agents, they are also capable of presenting foreign antigens and priming naïve T cells (6). These cells have been studied extensively in fishes and the specifics of their development and functional diversity (including development of M1 and M2 functional phenotypes) within teleosts have recently been covered in review articles (7, 8).

DCs were initially “baptized” by Ralf Steinman based on their morphology characterized by long, branching pseudopodia (9). A unifying characteristic of DCs is their superior capability to activate naïve T cells through presentation of MHCI and II-associated foreign antigens and costimulatory molecules on their surface (2, 10). Studies on mammalian DCs have been facilitated by development of protocols for *in vitro* generation of large numbers of DCs (11–14). Myeloid DCs can be generated *in vitro* following incubation of monocytes with GM-CSF and IL-4 for up to 5 days. This results in development of a relatively homogeneous population of immature DCs which require further activation with microbial products such as lipopolysaccharide, bacterial DNA, and double stranded RNA or cytokines such as TNF-α in order to achieve a mature state (15). The maturation of DCs is hallmarked with increased expression of MHCII and costimulatory molecules (e.g., CD80, CD86) on their plasma membrane and decreased ability to endocytose soluble antigens.

Piscine DC-like cells have been described *in vivo* in different teleost species including salmonids, zebrafish, and medaka (16–21); however, lack of protocols and tools for large-scale production of mature fish DCs *in vitro* has hampered further characterization of these cells. Homologs of most of the major cytokines and growth factors involved in activation and differentiation of various leukocyte types have been identified and isolated in fish. However, the essential growth factors—GM-CSF and FMS-like tyrosine kinase three ligand (FLT3L) used to differentiate mammalian DCs *in vitro* (11, 22) have not been identified in any fish species suggesting that orthologs of these genes may be absent below the level of tetrapods.

We have previously reported that 24 h *in vitro* stimulation of salmon mononuclear phagocytes with class B CpGs, a ligand for TLR9 and TLR21 (23, 24) upregulates a number of immune genes some of which are highly expressed in mature DCs (25). However, data about the effects of TLR ligands on antigen-presenting functions of piscine APCs are still scarce. In the current study, we have investigated the effects of long-term *in vitro* stimulation with TLR ligands, including CpG-B (2006PS) and polyI:C (a TLR3/22 ligand), on primary salmon MPs. The propensity of the CpG stimulation to induce differentiation of MPs as shown by appearance of cells with “dendritic” morphology prompted us to investigate the phenotypical and the functional characteristics of these APCs. We further discuss the capacity of the CpG stimulation to induce a proinflammatory, M1 transcriptional profile but an exhausted allostimulatory activity of salmon APCs.

## Materials and Methods

### Fish

Atlantic salmon (*Salmo salar*) strain Aquagen standard (Aquagen, Kyrksæterøra, Norway) was obtained from the Tromsø Aquaculture Research Station (Tromsø, Norway). The fish were kept at about 10°C in tanks supplied with running filtered water and were fed on commercial, dry food (Skretting, Stavanger, Norway). All experiments were approved by the national committee for animal experimentation (Norwegian Animal Research Authority) and performed according to its guidelines.

### Reagents

Phosphorothioate-modified CpG-B (5′-TCGTCGTTTTGTCGTTTTGTCGTT-3′) were purchased from Thermo Scientific and polyI:C from InvivoGen. Cy5-conjugated CpG-B was obtained from Eurogentech. The antibodies against the β-chain of salmon MHCII and TLR9 have been previously described (25, 26). The antibody against actin was purchased from Sigma Aldrich (A2066). CellTrace™ Violet, CellTrace™ CFSE, AlexaFluor546, and Ova-Alexa488, were purchased from Life technologies.

### Isolation of Leukocytes From Atlantic Salmon

HK and spleen leukocytes were isolated as described (27). The density of the leukocyte suspensions was adjusted to 7 × 10^6^ cells/ml and the cells were incubated in 6-well plates (21 × 10^6^ of cells/well) in L-15, supplemented with 0.1% fetal bovine serum (FBS) for 24 h before washing. In addition to displaying classical MP morphology, the adherent HK leukocytes were able to phagocytose large amounts of yeast cell wall particles (data not shown), endocytosed, and processed large amounts of ovalbumin (27) and expressed high levels of markers for MPs including the macrophage colony stimulator factor receptor (CSF-1R) and the macrophage scavenger receptor MARCO mRNAs (28). Adherent HK MPs (stimulators) and non-adherent spleen lymphocytes (responders) were washed and incubated in L-15 supplemented with 5% FBS, penicillin (10 U/ml), streptomycin (10 mg/ml) as described below.

### Mixed Leukocyte Reactions (MLRs)

The adherent HK MPs were either left non-stimulated or treated with 2 μM CpG-B for 24 h or 7 days and were harvested on ice using Ca/Mg-free PBS/100 mM EDTA. Stimulators and responders were stained with CellTrace™ CFSE and CellTrace™ Violet, respectively. Staining of stimulators with CFSE was used to distinguish them from proliferating responders with reduced CellTrace™ Violet staining. The cells were washed with PBS and stained with 2 μM dye at 10^6^cells/mL density for 10 min at room temperature. After staining, the cells were washed three times with L-15, 5% FBS using a 15 min interval between each washing. Responders were mixed with both non-stimulated and CpG-treated stimulators from the same or other individuals in duplicates at 2:1 ratio (1 × 10^5^/0.5 × 10^5^ cells) in 96-well round-bottom plates and incubated for 3, 5, 7, and 14 days before flow cytometry analysis. Fifty per cent of the medium was refreshed at 3-day intervals.

### Flow Cytometry

To investigate the surface expression of MHCII together with the Ova and CpGs uptake capacity, non-stimulated and CpG-stimulated (2 μM, 7 days) MPs were incubated with 10 μg/ml of Ova-Alexa488, or 2 μM CpG-Cy5 conjugates for 1 h. The cells were harvested on ice using Ca/Mg-free PBS/100 mM EDTA. The samples were washed with ice-cold PBS and incubated with a polyclonal salmon MHCIIβ antibody (1,000-fold dilution) for 1 h in PBS, 5% FBS on ice. The secondary Alexa546 goat anti-rabbit antibody was diluted to 1 μg/ml in PBS, 5% FBS and the cells were incubated for 30 min on ice prior to washing, and flow cytometry. Samples from the MLRs (described in the previous paragraph) were harvested and analyzed directly without staining. The cells were analyzed using FACSAria (Becton Dickinson). Statistical analysis was performed with Student's *t*-test.

### Confocal Microscopy

MPs cultured on 15 mm coverslips in 24-well plates cell culture plates (Falcon) were either left non-stimulated or treated with 2 μM CpGs for 7 days. The cells were then incubated with 2 μM CpG-Cy5 for 1 h, stained and analyzed as previously described (24).

### Microarray Analysis

MPs were stimulated with 2 μM CpGs and 20 μg/ml of polyI:C and sampled after 24 h and 7 days. Control cells were left non-stimulated. On day 6, Gardiquimod (1 μg/ml) was added to restimulate the cells and RNA samples were harvested from the restimulated and the non-restimulated cells on day 7. Cells from three individuals were stimulated in parallel. Gene expression was analyzed using the salmonid oligonucleotide microarray (SIQ2.0, NCBI GEO platform GPL10679) (29) consisting of 21 K features printed in duplicates on 4 × 44 K chips produced by Agilent Technologies (CA, USA). Two-color hybridizations were applied, where test samples labeled with Cy5 dye were competitively hybridized against control samples labeled with Cy3 dye per array. Concentration of total RNA was measured with NanoDrop 1,000 Spectrometer (Thermo Scientific, Waltham, MA, USA) and quality was assessed using Agilent 2100 Bioanalyzer with RNA Nano kits (Agilent Technologies). Samples with RNA integrity number (RIN) of eight or higher were accepted for microarray analyses. RNA from cells obtained from two individuals was pooled and used for the analysis. Unless specified otherwise, all reagents and equipment used for microarray analyses were from Agilent Technologies. RNA amplification and labeling were performed using Quick Amp Labeling Kits, Two-Color, and RNA Spike-In Kits, Two-Color following the manufacturer's protocol for 4 × 44 K microarrays; each reaction used 500 ng of total RNA. Gene Expression Hybridization Kit was used for fragmentation of labeled RNA. Hybridizations to microarrays were performed in hybridization oven (Agilent Technologies) at 65°C and rotation speed of 10 rpm. Arrays were washed with Gene Expression Wash Buffers 1 and 2 and scanned with a GenePix 4,100A (Molecular Devices, Sunnyvale, CA, USA). GenePix software was used for spot-grid alignment, feature extraction, and quantification. Assessment of spot quality was done with aid of GenePix flags. After filtration of low quality spots, Lowess normalization of log2-expression ratios was performed. The replicates showed close concordance of gene expression profiles (Pearson r = 0.93 ± 0.01). Features with >2-fold change in both samples per treatment were selected as differentially expressed.

### Cell Surface Protein Isolation and Mass Spectrometry Analysis

Twenty ml of HK leukocyte suspensions (7 × 10^6^/ml) were seeded in T75 flasks (BD Biosciences) and allowed to adhere overnight. Following washing, adherent MPs were further incubated without stimulation or treated with 2 μM CpGs for 7 days. Cells from two individuals with confluence of 80–90% were selected for isolation of cell surface proteins using Pierce® Cell Surface Protein Isolation Kit (Thermo Scientific) and following manufacturer's instructions. Briefly, the cells were rinsed with ice-cold PBS and cell surface proteins were labeled with 10 ml of Sulfo-NHS-SS-Biotin solution for 30 min at 4°C. Following a quenching reaction, the cells were collected with a scraper, washed with TBS, lysed with 500 μL of Lysis Buffer and subjected to low power sonication on ice. Biotinylated proteins in clarified supernatants were captured using NeutrAvidin Agarose at RT for 1 h. The proteins were eluted from the agarose beads using SDS sample buffer, 50 mM DTT at RT for 1 h and subjected to SDS PAGE and Coomassie staining. Gel bands of interest were excised and subjected to in-gel reduction, alkylation, and tryptic digestion using 6 ng/μl trypsin (V511A, Promega). Peptide mixtures containing 1% formic acid were loaded onto a nanoAcquityTMUltra Performance LC (Waters), containing a 3-μm Symmetry® C18 Trap column (180 μm × 22 mm) (Waters) in front of a 3-μm AtlantisTM C18 analytical column (100 μm × 100 mm)(Waters). Peptides were separated with a gradient of 5–90% acetonitrile, 0.1% formic acid, with a flow of 0.4 μl/min eluted to a Q-TOF Ultima Global mass spectrometer (Micromass/Waters), and subjected to data-dependent tandem mass spectrometry analysis. Peak lists were generated by the ProteinLynx Global server software (version 2.1), and the resulting pkl files were searched against the NCBInr 20090214 protein sequence database using MASCOT search engine (http://www.matrixscience.com/). Peptide mass tolerances used in the search were 100 ppm, and fragment mass tolerance was 0.1 Da.

### Western Blot Analysis (WB)

The flow-through lysates which contain proteins that did not bind to Neutravidin and LDS eluates, enriched in biotinylated proteins were analyzed as previously described (25). Briefly, samples dissolved in 4 X LDS were run on NuPAGE Novex Bis-Tris 4–12% gels (Invitrogen) in MOPS running buffer. The proteins were transferred to PVDF membranes, blocked (Tris-buffered saline, 5% BSA, 0.1% Tween-20) for 1 h, and incubated overnight with primary Abs (1: 1000 dilution for MHCII and 1:100 for the actin antibody) followed by 1 h of incubation with the secondary HRP-conjugated antibodies (1: 10 000 dilution). The blots were developed with SuperSignal West Pico substrate (Pierce, Rockford, IL, USA).

## Results

### Prolonged *in vitro* Stimulation With CpGs Leads to Morphological Differentiation of Salmon MPs and Development of Cells With “Dendritic” Morphology

**Figure 1:**
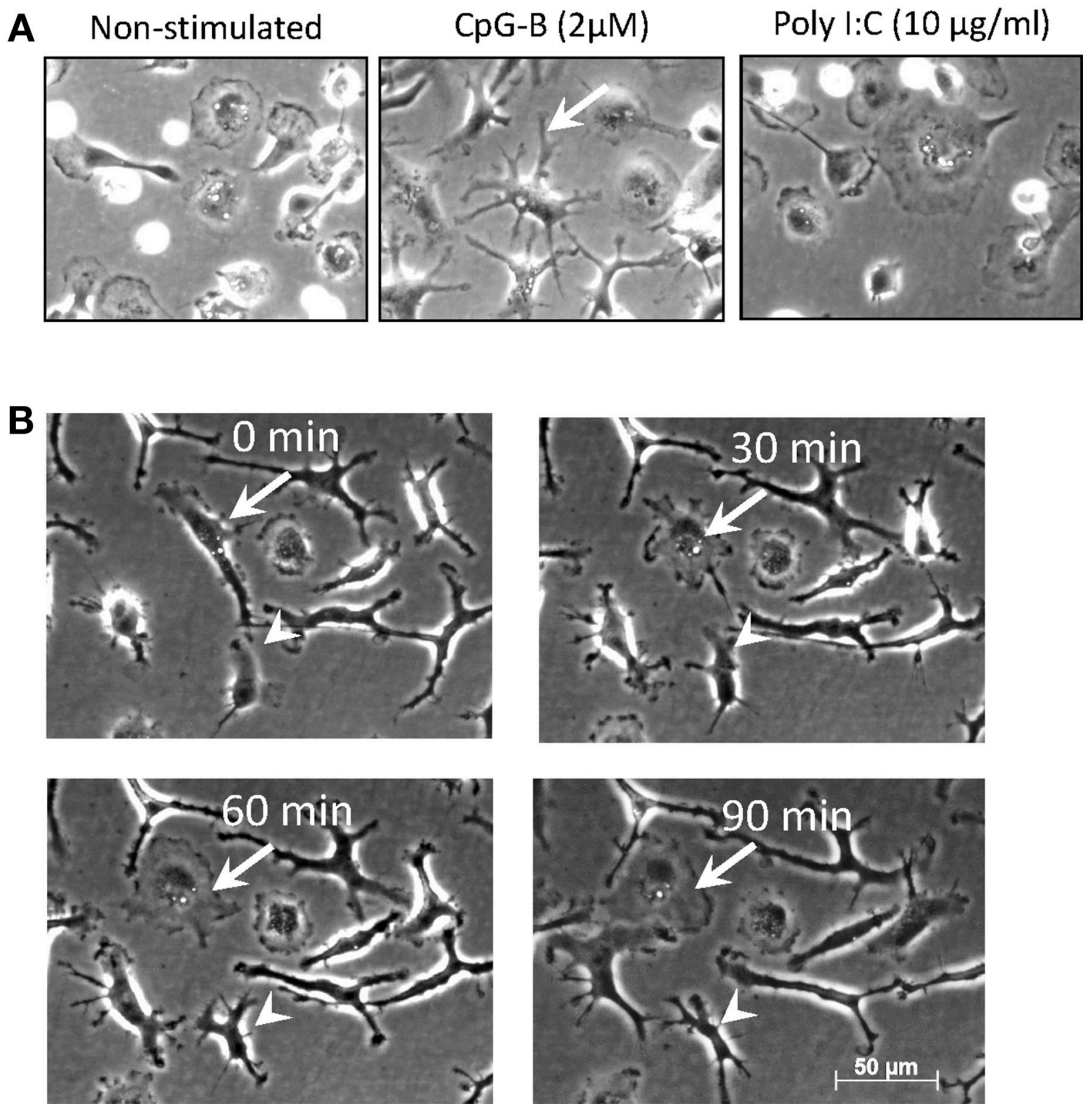
Salmon MPs develop dendritic morphology following prolonged treatment with CpGs. **(A)** Images of non-stimulated MPs and cells stimulated for 7 days with 2 μM CpGs and 20 μg/ml of polyI:C. The arrow indicates a typical DC-like cell observed in the CpG-treated samples. **(B)** Dynamic reorganization of the morphology of CpG-treated MPs. Cells were stimulated as in panel A and images were taken at 30min intervals over a period of 90min. The arrow points at elongated DC-like cell which changes morphology into a more rounded macrophage-like cell. The arrowhead indicates a cell which undergoes the opposite changes. Images were taken at X200 magnification.

When adherent salmon head kidney (HK) MPs were stimulated *in vitro* for >5 days with CpGs (2 μM) many of the cells developed relatively long, branching pseudopodia, a morphological feature manifested by DCs (9) and, in some cases, M1 macrophages (30) (Figure 1A). This was not observed when cells were stimulated in parallel with polyI:C (20 μg/ml). After 7 days of treatment with CpGs, cells that had the original macrophage-like morphology were also present. A time lapse imaging showed that while many of the cells retained their dendritic-like morphology, the shape of some of the CpG-stimulated cells was dynamic as they could transition between dendritic-like to macrophage-like and vice versa morphology within a time span of 90 min (Figure 1B).

### CpGs Upregulate Allostimulatory Activity and Surface MHCII Expression in Salmon MPs After a Short but Not After a Prolonged Treatment

**Figure 2:**
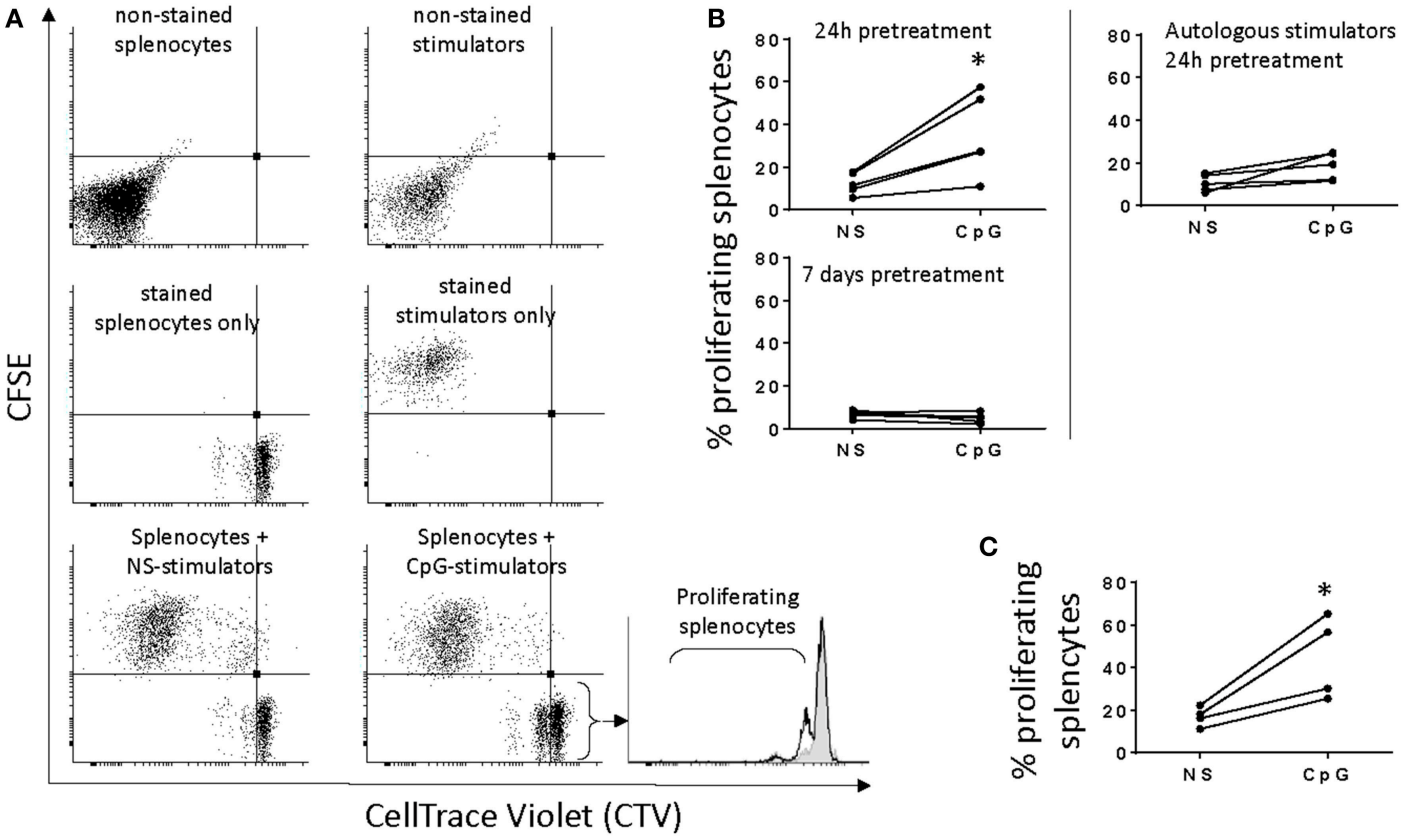
Short, but not prolonged treatment of salmon MPs with CpGs upregulates their allostimulatory activity. **(A)**, adherent HK MPs were stained with CFSE upon 24 h of stimulation with 2 μM CpGs. Splenocytes stained with CellTrace Violet (CTV) were used as responders. The dot plots show that a significant number of stimulators take up CTV which necessitates their staining with CFSE in order to distinguish them from proliferating responders with reduced CTV staining. The CFSE-negative events were gated and displayed in histograms to quantify the percentage of proliferating splenocytes with reduced CTV staining. In the representative histogram (bottom right), responders were cultured with non-stimulated (NS) MPs from another individual (filled gray area), and stimulators pretreated with CpGs for 24 h (black contour). **(B)**, Stimulators pretreated with CpGs for 24 h but not 7 days show increased allostimulatory capacity (*n* = 5). Autologous stimulators did not significantly activate splenocyte proliferation even after pretreatment with CpGs for 24 h (*p < 0.05). **(C)**, Direct CpG stimulation of responders was included as a positive control for cell proliferation (*n* = 4). In all of the experiments, the responders were cultured *in vitro* for 15 days.

In order to estimate the allostimulatory capacity of non-stimulated MPs and cells stimulated with CpGs for 24 h and 7 days we performed MLRs with spleen lymphocytes from different individuals. The experimental conditions were chosen based on preliminary experiments using responders from spleen, HK, and blood (results not shown). Spleen lymphocytes depleted of adherent cells showed the highest response and were further used in the study. Using a CellTrace™ Violet Cell Proliferation Kit we found that unlike autologous MPs, stimulators from other individuals incubated with responder spleen lymphocytes for 2 weeks induced modest spleen cell proliferation of the total population as indicated by the increased percentage of responders with reduced amount of dye. No significant response was observed in MLRs incubated for 3, 5, and 7 days (data not shown). The analysis setup of the flow cytometry data is shown in Figure 2A—proliferating responders are located in the lower left quadrant of the dot plots due to reduced CTV staining while CFSE staining was used to gate out stimulators. Incubation of splenocytes with stimulators from other individuals that had been stimulated with CpGs for 24 h induced significant proliferation response (*p* < 0.05), whereas autologous stimulators and MPs stimulated with CpGs for 7 days prior to MLRs did not induce significant splenocyte proliferation (Figure 2B). CpG-B have been previously shown to be a potent mitogen for salmon splenocytes (31) and in the current study direct stimulation of responders with CpGs for 2 weeks was used as a positive control for the proliferation assay (Figure 2C). The CpG-induced proliferation of salmon leukocytes requires relatively high concentrations of CpGs (>0.5 μM) (31). This, as well as the fact that autologous stimulators treated with CpGs for 24 h and MPs stimulated for 7 days prior to MLRs did not induce significant splenocyte proliferation, indicates that the observed MLR response was not due to carryover of residual CpGs. In all of the experiments, the responders were cultured *in vitro* for the same period (15 days).

**Figure 3:**
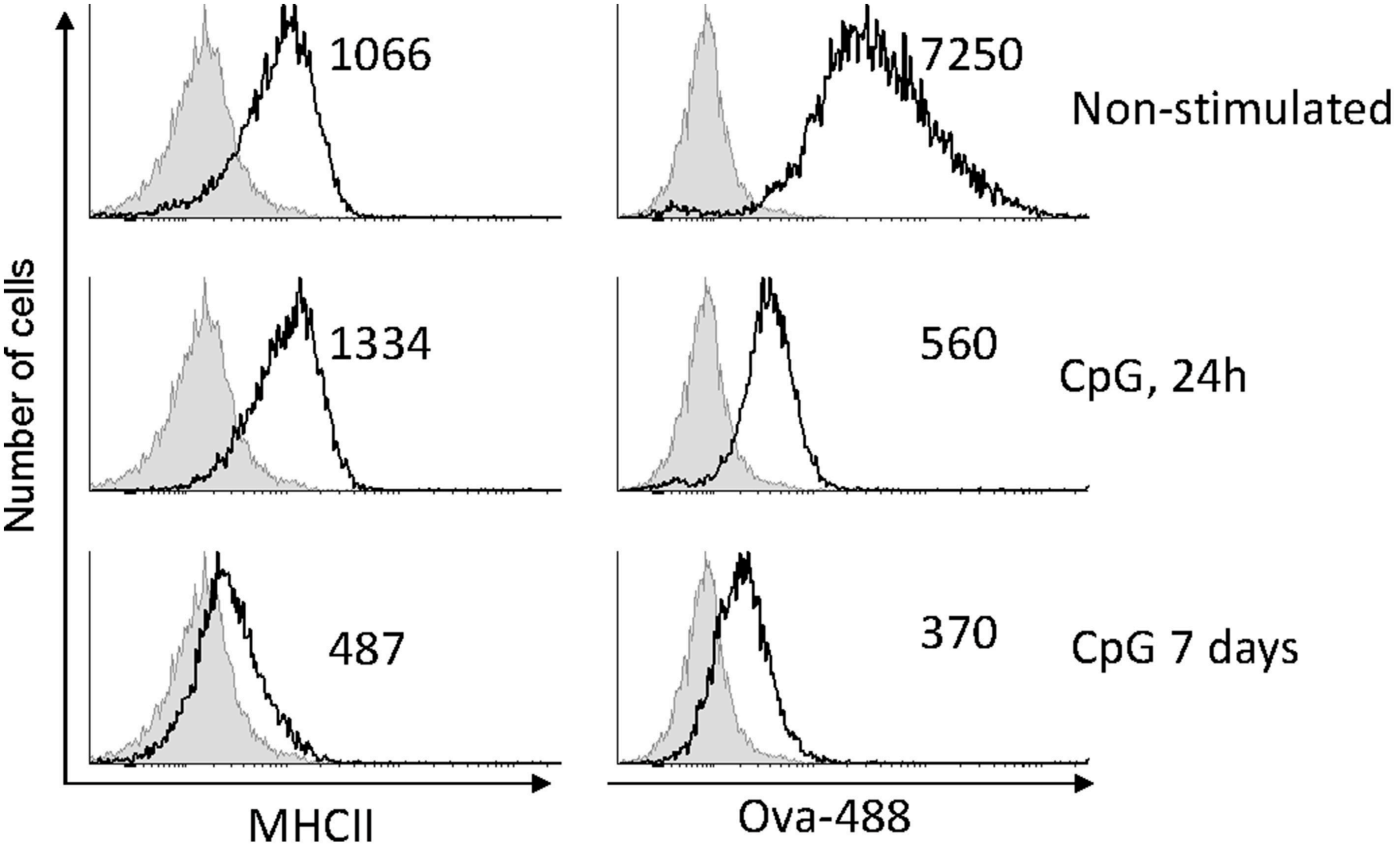
CpG stimulation reduces antigen uptake capacity of MPs and, after a prolonged treatment, downregulates surface MHCII expression. MPs were either left untreated or stimulated with 2μM CpGs for 1 and 7 days, as indicated. The cells were incubated with 10 μg/ml of Ova conjugated with AlexaFluor-488 for 1 h prior to harvesting and analysis using flow cytometry. The numbers in the individual histograms show the mean fluorescence intensities of the stained samples (black contours). The non-stained samples are represented with filled gray contours. Representative results from two experiments with cells from different individuals are shown.

Using flow cytometry, the surface expression of MHCII and the level of Ova uptake were measured and compared between CpG-stimulated and non-treated HK-derived MPs. The representative results shown in Figure 3 demonstrate that there was a slight increase of MHCII expression 1 day after the cells were exposed to CpGs, while on day 7 the surface expression on MHCII was substantially reduced. The uptake of fluorescent Ova was reduced on day 1 and was even lower on day 7.

**Figure 4:**
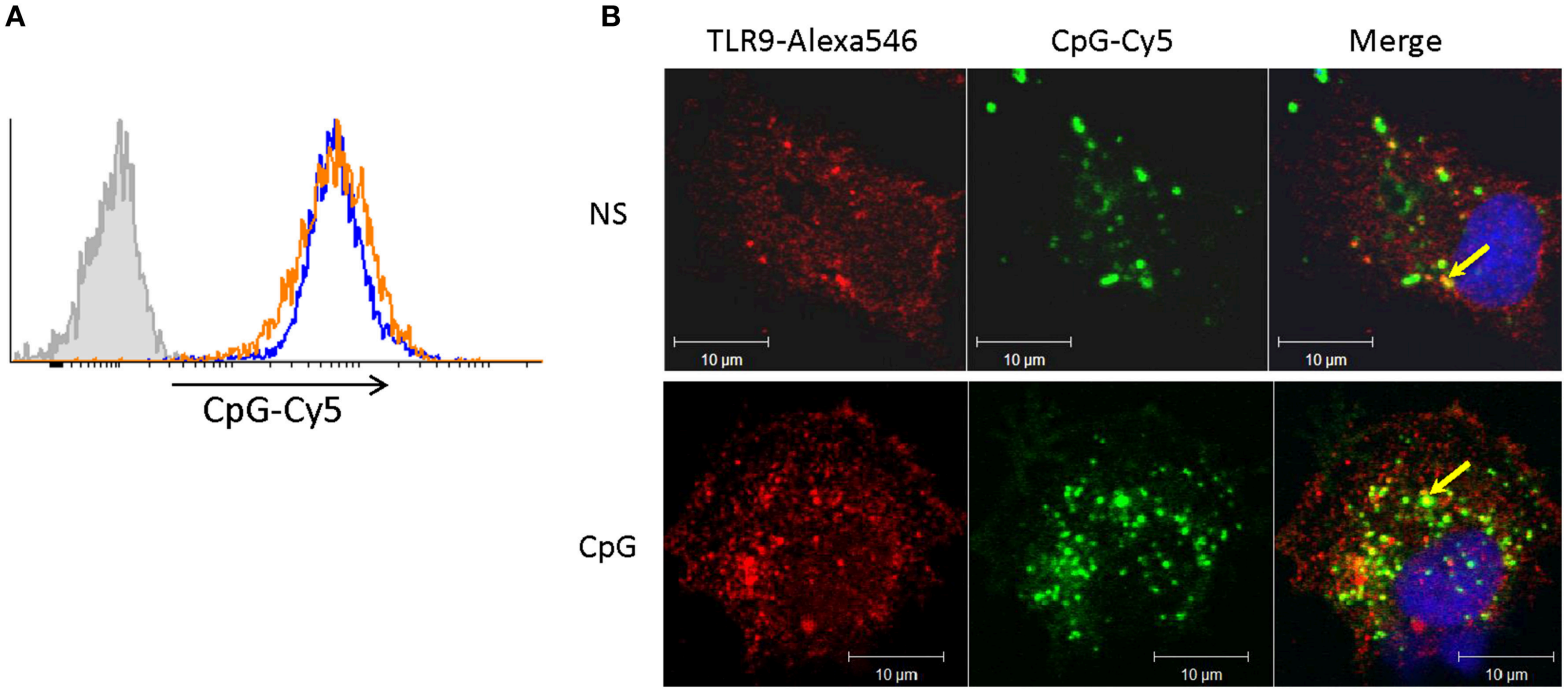
Cells stimulated with CpGs for 7 days retain capacity to take up CpG ODNs and to translocate them into TLR9-positive endocytic compartments. **(A)** MPs were either left non-stimulated (blue contour) or were treated with 2 μM CpGs for 7 days (orange contour) prior to incubation with fluorescent (CpG-Cy5) ODNs for 1 h and flow cytometry analysis. The filled gray contour represents cells incubated without fluorescent CpGs. **(B)** Non-stimulated cells (NS) and cells pretreated with CpGs for 7 days were incubated with CpG-Cy5 for 1 h prior to fixation, permeabilization, and staining of intracellular TLR9. The endocytosed CpGs were visualized in the far-red channel and are shown in green pseudocolor. The nuclei were stained with SYTOX Green (blue pseudocolor). The colocalization between TLR9 and CpG-positive vesicles (yellow color) is indicated with arrows in the merged images.

In contrast to Ova endocytosis, the uptake of CpG ODNs was not affected in cells pre-treated with CpGs for 7 days. In the experiment presented in Figure 4 cells were treated with CpGs for 7 days and then incubated with fluorescent CpG-Cy5 ODNs for 1 h prior to flow cytometry (Figure 4A) and immunostaining/confocal microscopy (Figure 4B). The results demonstrate that neither the level of uptake of CpGs ODNs nor the ability to accumulate the ODNs within TLR9-positive intracellular compartments was affected in the CpG-treated cells.

### Transcriptional Response to Long-Term CpG and PolyI:C Stimulation. CpG-Pretreated Salmon MPs Retain Ability to Upregulate Proinflammatory Genes Upon Restimulation With Gardiquimod

**Figure 5:**
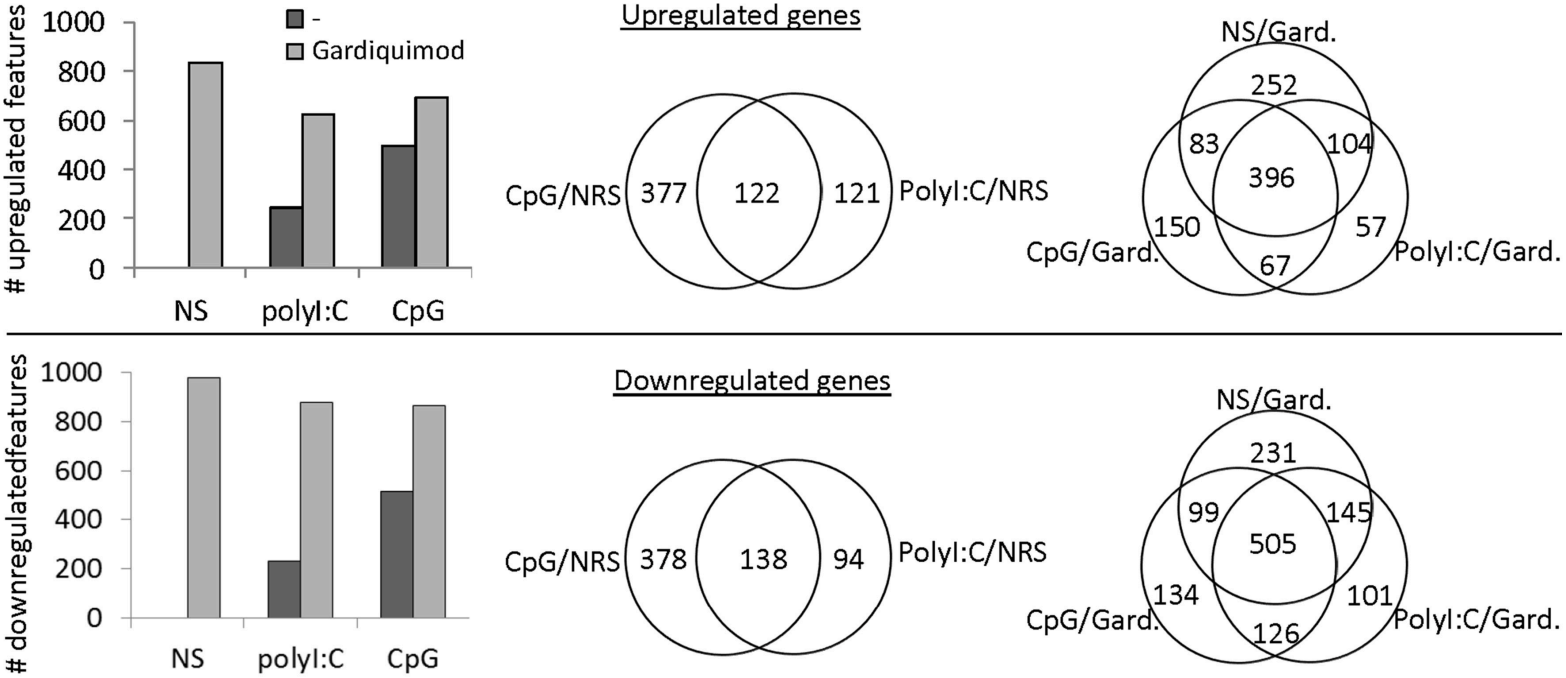
Microarray analysis—transcriptional response of salmon MPs to long term stimulation with polyI:C and CpGs and restimulation with gardiquimod. MPs were left non-stimulated (NS) or stimulated with 2 μM CpGs or 20 μg/ml of polyI:C. After 6 days, samples were restimulated with 1 μg/ml of Gardiquimod and RNA from both non-restimulated (NRS) and restimulated samples was sampled on day 7. Pooled RNA samples of cells from two individuals were analyzed using a salmonid oligonucleotide microarray. Features with fold change values of +2 and −2 as compared to the NS samples were considered up- and downregulated, respectively. The histograms show the total numbers of up- and downregulated features in the different samples. The Venn diagrams show the numbers of common and unique up- and downregulated genes among the samples.

**Figure 6:**
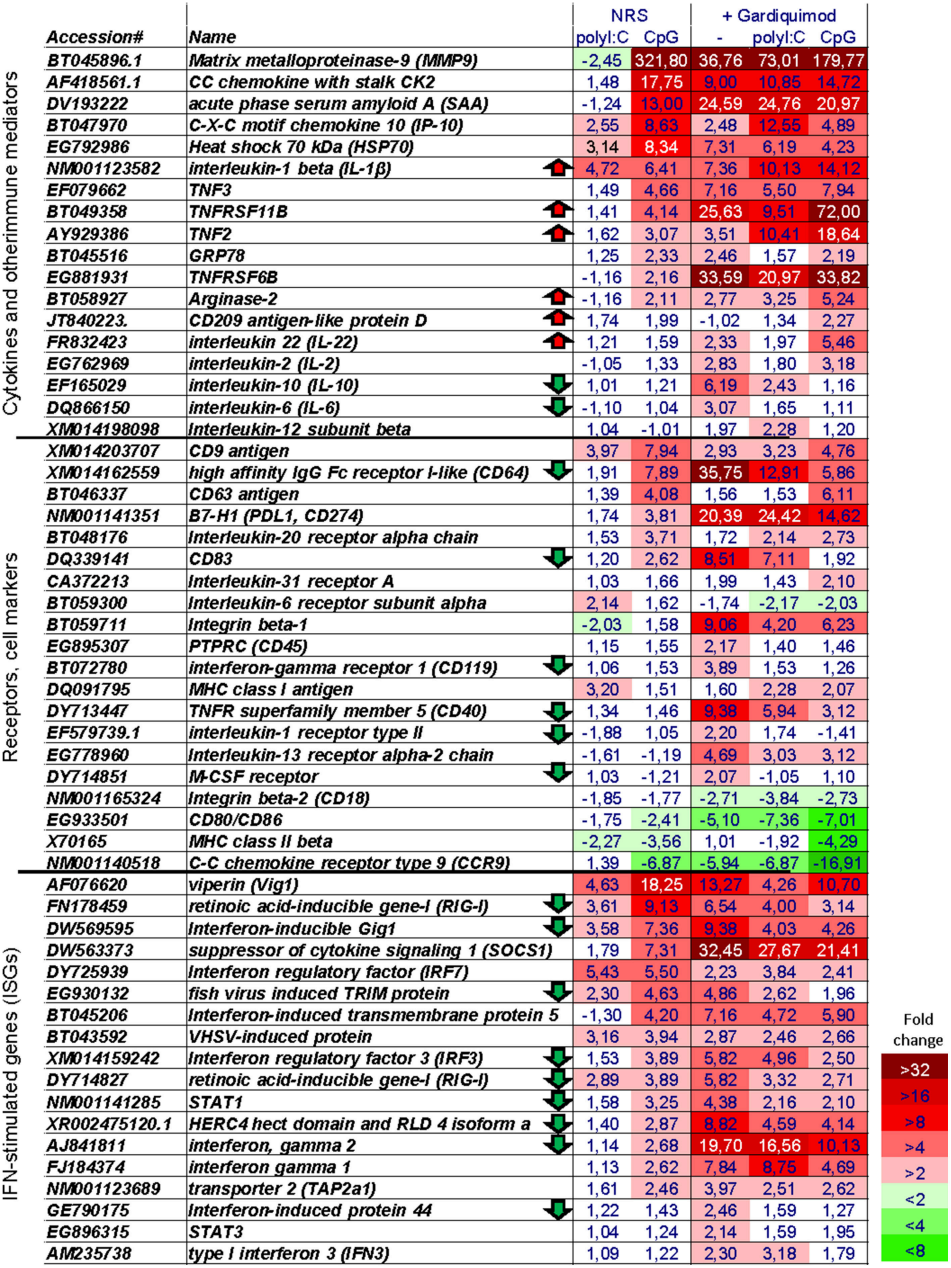
Microarray heat map showing the expression of selected immune genes. The numbers show the “fold change” values as compared to non-stimulated cells. The heat map legend is shown in the lower right corner. Note, that genes of interest have been tentatively assigned to three groups; however, many of them can be assigned to more than one group. Arrows indicate genes whose expression is at least 1.9-fold higher or lower in CpG + Gardiquimod samples as compared to samples stimulated only with Gardiquimod.

The differentiation and maturation of APCs is a complex process in which the cells undergo a thorough transcriptomic reprogramming (32). Here, using a microarray platform we have analyzed the transcriptional response of salmon MPs to the long-term stimulation with CpGs and polyI:C. The latter, unlike CpGs, did not induce similar morphological differentiation of salmon MPs although it had a superior capacity to induce upregulation of IFN-stimulated genes (*ISGs*) after 24 h of stimulation (Figure S1). The results from the microarray analysis demonstrated that, after 7 days of stimulation, CpGs both up- and downregulated larger number of genes as compared to the polyI:C treatment (Figure 5). Unlike polyI:C, the CpG treatment upregulated proinflammatory cytokines (*IL-1*β*, TNF2/3*), genes associated with inflammation (including *SAA* and *MMP9*) and secreted TNF receptor superfamily (*TNFRSF*) members—*TNFRSF11B* and *TNFRSF6B* (Figure 6, Figure S2).

Among genes known to be implicated in the antigen-presenting functions of APCs, the CpG stimulation upregulated *CD83*, and the inhibitory *B7-H1* (*PDL1, CD274*) molecule while it downregulated the expression of *MHCII, CD80/86*, and *CCR9* homologs (Figure 6, Figure S2).

Interestingly, although, as mentioned above, after 24 h of stimulation, polyI:C upregulated ISGs to a much higher extent as compared to CpGs, after 7 days of stimulation, the opposite was observed for many key ISGs such as *CXCL10* (*IP-10*), *Vig1, Mx, SOCS1, STAT1*, and *RIG-I* as well as type II *IFN* homologs (Figure 6, Figure S2).

After 6 days of stimulation, the cells were restimulated with Gardiquimod (a TLR7/8 ligand) in order to investigate if the CpG- and polyI:C-treated cells retained their potential to respond to stimulation with TLR ligands. Compared to non-pretreated cells, Gardiquimod restimulation up- and downregulated fewer genes in CpG and polyI:C pretreated cells (Figure 5) indicating that the pretreatment modulated but did not completely suppress the potential of the cells to respond to secondary stimulations with TLR ligands. Figure 6 shows the expression of selected groups of immune genes including cytokines and other immune mediators, receptors, and cell markers and ISGs. Upregulation of many of the selected genes was suppressed by the CpG and, to a lower extent, by the polyI:C pretreatments. Some of the genes whose upregulation was not repressed and was further upregulated in CpG- pretreated cells included proinflammatory cytokines (*IL-1*β, *IL-22*, and *TNF2*), and secreted TNF receptor family members (*TNFRSF11B* and *TNFRSF6B*). Interestingly, many ISGs were among the genes whose upregulation was suppressed by the pretreatment including *RIG-I, IFN-inducible Gig1, IRF3, STAT1*, as well as *IFN*-γ. Many of the genes listed in Figure 6 can be assigned to more than one of the three categories. Therefore, it should be mentioned that the expression of immune receptors and cell markers, including CD64, CD83, CD40, and the M-CSF receptor, is known to be regulated by IFNs (33–36) and their gardiquimod-induced upregulation was also suppressed in CpG-pretreated cells.

The microarray data was validated using real-time PCR analysis in which samples from three individuals were analyzed separately. As demonstrated in Figure S2, except for the lower values, a commonly observed phenomenon caused by the lower dynamic range of the microarray analysis as compared to that of the real-time PCR, the two types of analysis produced similar results. The sequences of the primers used in the real-time PCR analysis are shown in Table S1.

### Identification of Differentially Expressed Cell Surface Proteins on Non-stimulated and CpG-Treated Salmon MPs

**Figure 7:**
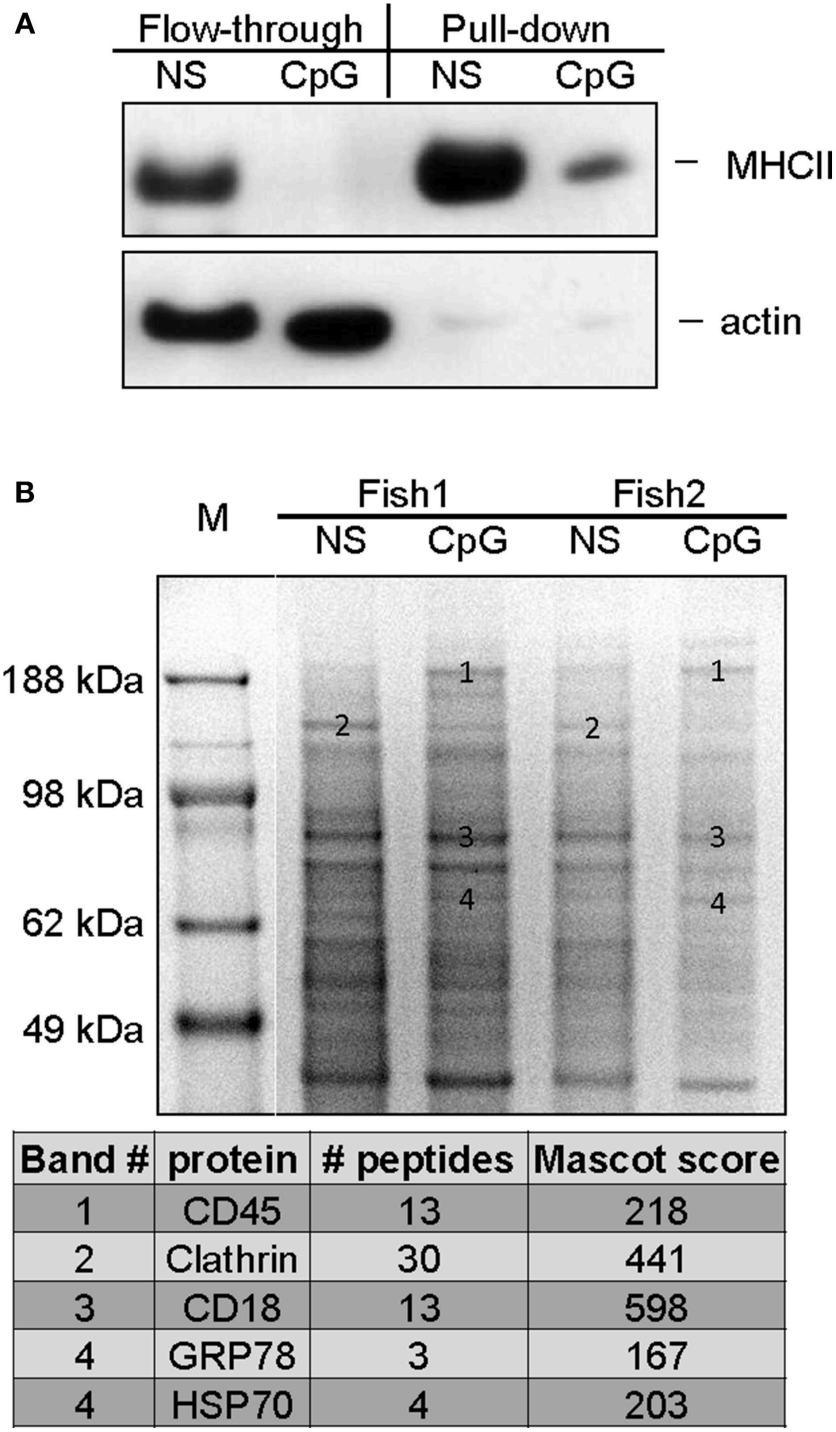
Mass spectrometry analysis of cell surface proteins of non-stimulated and CpG-treated salmon MPs. The cells were either left non-stimulated or were stimulated with CpGs for 7 days. Cell surface proteins were labeled with biotin and purified as described in Materials and methods. **(A)** The efficiency of the cell surface protein purification was confirmed with WB analysis. Flow-through and pull-down samples enriched in proteins associated with cell surface from control non-stimulated and CpG-stimulated cells were run on SDS PAGE and protein levels of MHCII-β and actin were detected with polyclonal antibodies. **(B)** Samples enriched in cell surface proteins isolated from cells of two individuals were run in parallel on SDS-PAGE, and stained with Coomassie. Bands of interest were excised, and subjected to MS/MS analysis. The table below the gel image lists identified proteins in bands whose ID numbers are indicated on the gel image.

To further define features that are characteristic for CpG-treated MPs, expression of surface proteins upon CpG-treatment were examined by mass spectrometry and compared to non-treated cells. Samples acquired using the cell surface protein isolation kit were analyzed with WB in order to estimate the efficiency of the protein purification. Compared to the flow-through lysates the pull-down samples are expected to be depleted of proteins which are not directly associated with the plasma membrane. The results shown in Figure 7A demonstrate that compared to the flow-through samples, the pull-down samples contained more MHCII while the actin levels were barely detectable, thereby confirming the efficiency of the cell surface isolation procedure.

Major SDS PAGE protein bands present in samples of cell surface proteins from non-stimulated MPs and cells treated with CpGs for 7 days were isolated and analyzed using tandem mass spectrometry (MS/MS). Samples from cells of two individuals were run in parallel and consistently regulated bands were selected for the analysis (Figure 7B). The analysis identified CD45 in a high molecular weight band that was upregulated by the CpG treatment. Another high molecular weight band which appeared to be downregulated in CpG-treated samples contained clathrin whereas CD18, an integrin involved in cell adhesion, was identified in a major band that did not seem to be regulated by the treatment in any of the two analyzed individuals. The HSP70 family members GRP78 and HSP70 were identified in bands which appeared to be slightly upregulated by the CpG treatment.

## Discussion

In the current study, long-term *in vitro* stimulation (>5 days) with CpGs induced functional and phenotypical differentiation of salmon MPs. Although these cells developed dendritic morphology and an M1-like proinflammatory transcriptional profile, they downregulated their surface MHCII expression, and had an impaired allostimulatory capacity.

The differentiation of MPs into cells with dendritic morphology appeared to correlate with upregulation of proinflammatory genes since cells stimulated in parallel with polyI:C did not exhibit similar morphological changes and had fewer inflammatory mediators upregulated after 7 days of stimulation. At the same time, the polyI:C-stimulated cells had some IFN-inducible genes upregulated at levels compared to, or even exceeding those in CpG-stimulated cells.

The term “dendritic cell” was introduced by Ralf Steinman, reflecting the specific morphology of DCs isolated from peripheral lymphoid organs (9). However, it should be noted that, under specific conditions, macrophages stimulated with LPS and IFN-γ may also adopt a similar morphology (30), characterized by long, branching pseudopodia. Therefore, considering all of the data we have obtained in this study, it would be more pertinent to conclude that the salmon MP cultures stimulated for 7 days with CpGs were enriched in activated M1 macrophages rather than DCs.

As with other types of primary cell cultures, the starting cultures of adherent salmon leukocytes were not homogenous and, most likely, included monocytes and macrophages at different developmental stages. However, the observation that the cells concertedly downregulated MHCII expression and the Ova uptake capacity suggests that the CpG-induced differentiation of these cells was relatively synchronized.

Similar to mammalian DCs, the shape of the CpG-stimulated salmon MPs was quite dynamic as the dendrites retracted and expanded in different directions within minutes. As speculated elsewhere, this property likely contributes to the antigen presenting function of DCs by allowing the APCs to scan a larger area and facilitate the capture of antigens (37). Regardless of the morphological differentiation, in our study, the CpG treatment downregulated the ability of the MPs to endocytose ovalbumin as soon as 24 h and even further at 7 days poststimulation. The fact that the capacity of the cells to take up CpGs was not attenuated indicates that the CpG stimulation affected only specific endocytic pathways, likely mannose receptor- or scavenger receptor-mediated endocytosis.

Upon maturation, DCs upregulate surface MHCII but lose their ability to take up and process soluble antigen (38, 39). In the current work, the downregulation of the capacity of CpG-stimulated MPs to take up ovalbumin may be considered as an indication that these APCs underwent a similar process of maturation. However, the transient upregulation followed by downregulation of surface MHCII expression after 7 days of treatment with CpGs indicated that during cultivation, the cells developed an exhausted or tolerogenic phenotype which was confirmed by the loss of their allostimulatory activity. It is not likely that this was due to reduced viability or a general loss of functionality since the cells retained ability to take up CpGs and to accumulate them in TLR9-positive endocytic compartments. In addition, although the cells responded to restimulation with Gardiquimod by upregulating fewer genes as compared to cell that had not been pretreated, they were still able to upregulate expression of a number of proinflammatory mediators. This indicates that the CpG-pretreated salmon MPs are not exhausted *per se* but, as reported elsewhere, might have been reprogrammed to respond differently to secondary stimuli (37).

Obviously, the MHCII downregulation was due to suppression of MHCII biosynthesis in CpG-stimulated MPs since the MHCII-β protein levels were downregulated not only on the cell surface but also in the whole lysates as demonstrated by the WB analysis. Similarly, stimulation of mouse peritoneal macrophages with CpGs downregulated the biosynthesis and the surface expression of MHCII (40). However, in that study the MHCII downregulation was observed after a relatively short stimulation (18 h) and, unlike in the current work, the treatment did not suppress the antigen uptake capacity of murine macrophages.

It has been shown that human monocytes differentiated into DCs with GM-CSF and IL-4 in the presence of TLR ligands develop a tolerogenic phenotype (41). This was due to downregulation of surface MHCII and upregulation of B7-H1, a suppressor of T-cell activation and a marker for tolerogenic DCs (42). Likewise, we found that the mRNA of *B7-H1* was upregulated in CpG-treated MPs, while the costimulatory molecule *CD80/CD86* was, on the contrary, downregulated. Furthermore, *TNFRSF6B*, a secreted TNF decoy receptor which is known for its capacity to induce tolerogenic phenotype in DCs and macrophages (43, 44) was also upregulated in these cells. Since in the current study, there was no concurrent treatment with GM-CSF, we cannot claim that we have obtained tolerogenic DCs; nevertheless, our data indicates that at least some of the mechanisms that control the differentiation and the maturation of professional APCs in presence of TLR ligands are conserved between teleosts and mammals.

Gardiquimod is a TLR7/8 ligand and, in the current study, it induced high expression of both proinflammatory genes as well as IFNs and IFN-stimulated genes (ISGs). As mentioned above, in cells pretreated with CpGs and polyI:C, Gardiquimod upregulated fewer genes as compared to non-pretreated cells. Furthermore, the level of upregulation of many genes in the former samples was generally lower as compared to the latter. A possible explanation for this is that the effect of Gardiquimod was likely influenced by autocrine factors. In this regard, it is possible that the attenuated induction of many of the genes we have observed might be due to modulation of IFN and cytokine receptor signaling. For example, *SOCS1*, which was highly upregulated in the CpG–treated cells inhibits the signaling initiated by type I and II IFN as well as cytokines which signal through the common gamma chain subunit of the interleukin (IL)-2 receptor (45). A strong negative regulatory activity on IFNa1 and IFN-γ signaling has also been exhibited by Atlantic salmon SOCS1 (46). It has been found that *SOCS1* expression can be upregulated directly by CpGs through a STAT-independent mechanism, a process which resulted in suppression of IFN-γ-, IL-6-, and GM-CSF-induced STAT1 phosphorylation and prevented the upregulation of MHCII in murine macrophages (47). Furthermore, it has also been demonstrated that TLR-induced SOCS1 expression in DC precursors leads to a blockade of DC differentiation (48).

Another factor that might have been involved in attenuated gene upregulation by autocrine factors is CD45. This is a protein tyrosine phosphatase receptor which, like SOCS1, is involved in negative regulation of cytokine and IFN receptor signaling (49). In the current study, the protein analysis indicated that the CD45 protein was highly expressed on the surface of CpG-treated cells since the molecule was identified in a high molecular weight band upregulated by the CpG stimulation. The Gardiquimod restimulation induced expression of both type I *IFN* and *IFN*-γ and therefore, the attenuated upregulation of ISGs in CpG-pretreated cells might be, at least in part, due to the inhibitory action of proteins such as SOCS1 and CD45 on the autocrine cytokine signaling. On the other hand, the high expression of ISGs in CpG-treated cells may be explained with elevated expression of IFN-regulatory factors (IRFs), such as *IRF3* and *IRF7* which themselves are ISGs. As we have already shown, overexpression of transgenic salmon *IRF3* and *7* in different salmonid cell lines is able to activate ISRE-dependent promoter elements which are essential for the induction of ISGs (50).

In addition to CD45, the surface protein analysis identified clathrin heavy chain in a down-regulated band and GRP78 and HSP70 in a band that appeared to be upregulated by the CpG treatment. Clathrin is a major protein involved in receptor-mediated endocytosis (51) and its downregulation might be related to the suppression of the antigen uptake capacity in CpG-treated cells. GRP78 and HSP70 are both members of the family of the HSP70 heat shock proteins and function in the endoplasmic reticulum as molecular chaperones. In addition, it has been found that these proteins are secreted and have been implicated in suppression of the allostimulatory capacity of APCs (52, 53). Therefore, HSP70 and GRP78 may have contributed to the reduced allostimulatory capacity of salmon MPs stimulated with CpGs. The downregulation of clathrin and the binding of HSP70 and GRP78 to the surface of CpG-treated salmon MPs need to be confirmed using specific antibodies and might be an interesting objective for future studies.

In summary, CpGs induced *in vitro* differentiation of salmon MPs into cells with dendritic morphology, an M1 transcriptional profile but exhausted allostimulatory phenotype and functions. These findings demonstrate that, despite the poor phylogenetic conservation of the growth factors involved in the differentiation of DCs, the major processes that orchestrate the effects of TLR ligands on the development of APCs are conserved between teleosts in mammals. These data also emphasize on the fact that the potent immunostimulatory properties of a TLR ligand would not necessarily translate into enhanced APC functions and highlight the complexity of the activation of fish immune cells by TLR ligands which may be used as potential adjuvants in the aquaculture.

## Data Availability

The microarray data presented in this publication has been deposited in the NCBI's Gene Expression Omnibus (GEO, https://www.ncbi.nlm.nih.gov/geo/) and is available under the accession number GSE126993.

## Author Contributions

DI designed, performed experiments, analyzed data, and prepared the manuscript. JJ participated in experimental design, data analysis, and manuscript writing. LL and HT were involved in cell culture experiments and assisted with manuscript preparation. AK and SJ designed and performed the microarray analysis and assisted with data analysis, and manuscript preparation.

### Conflict of Interest Statement

HT is currently employed by Vaxxinova Norway. AK and SJ were employed at Nofima Marin AS. The remaining authors declare that the research was conducted in the absence of any commercial or financial relationships that could be construed as a potential conflict of interest.
