# Supplementary material for: CpGs Induce Differentiation of Atlantic Salmon Mononuclear Phagocytes Into Cells With Dendritic Morphology and a Proinflammatory Transcriptional Profile but an Exhausted Allostimulatory Activity
Source: Front Immunol. 2019 Mar 13;10:378. doi: 10.3389/fimmu.2019.00378 (PMC6424866; doi:10.3389/fimmu.2019.00378)
Supplement: Supplementary file 1 [file Data_Sheet_1.PDF]

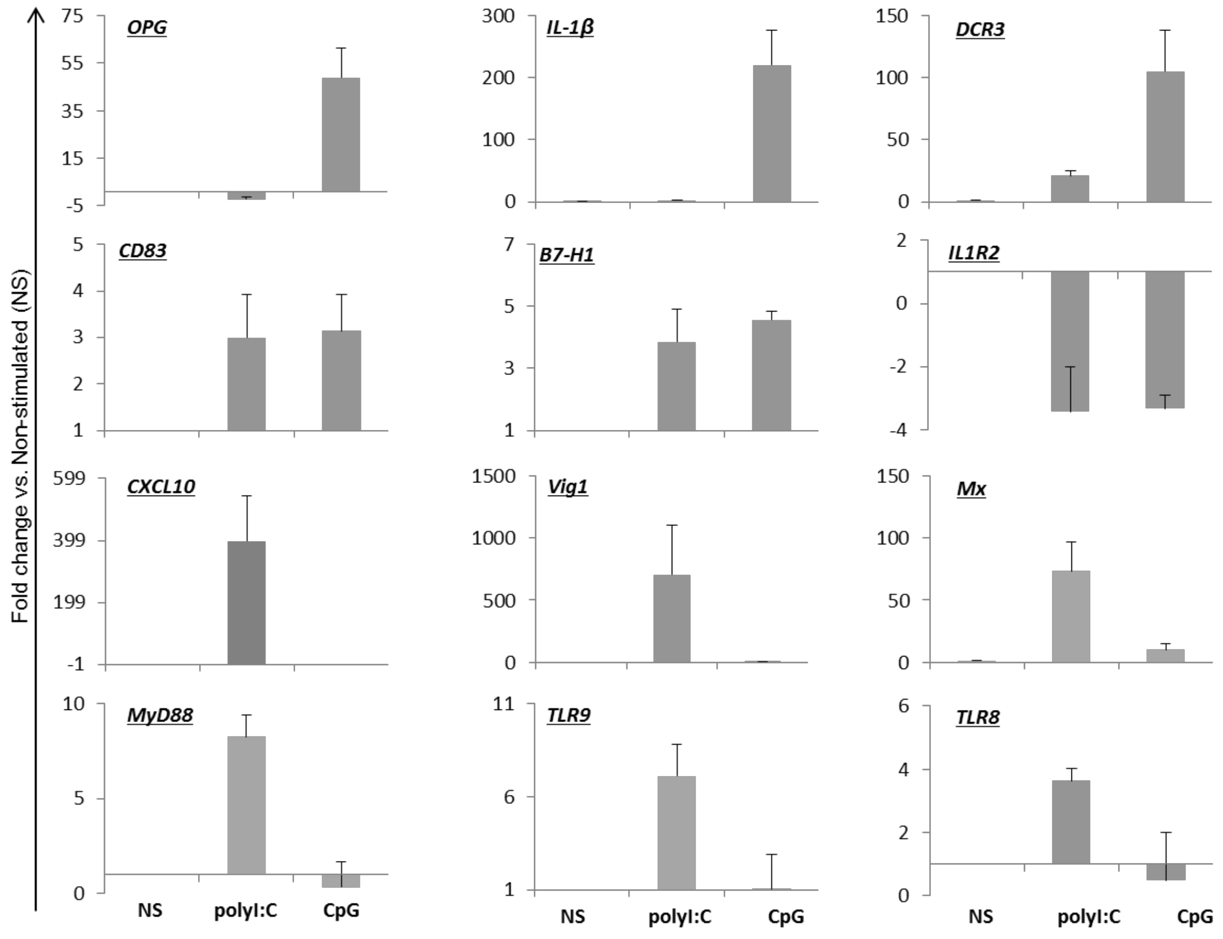


**Figure S1**. Real-time PCR analysis of gene expression in salmon MPs. The same cell preparations used for the microarray analysis were used; however, samples were harvested after 24h of stimulation. *EF1aB* expression was used as endogenous control and the data is presented as fold difference values as compared to the NS samples. The error bars show the standard deviation (N=3). The reaction conditions and data analysis were previously described (Front Immunol 2013. 4: 137). The primer and probe sequences and the type of the reactions are listed in Table S1.

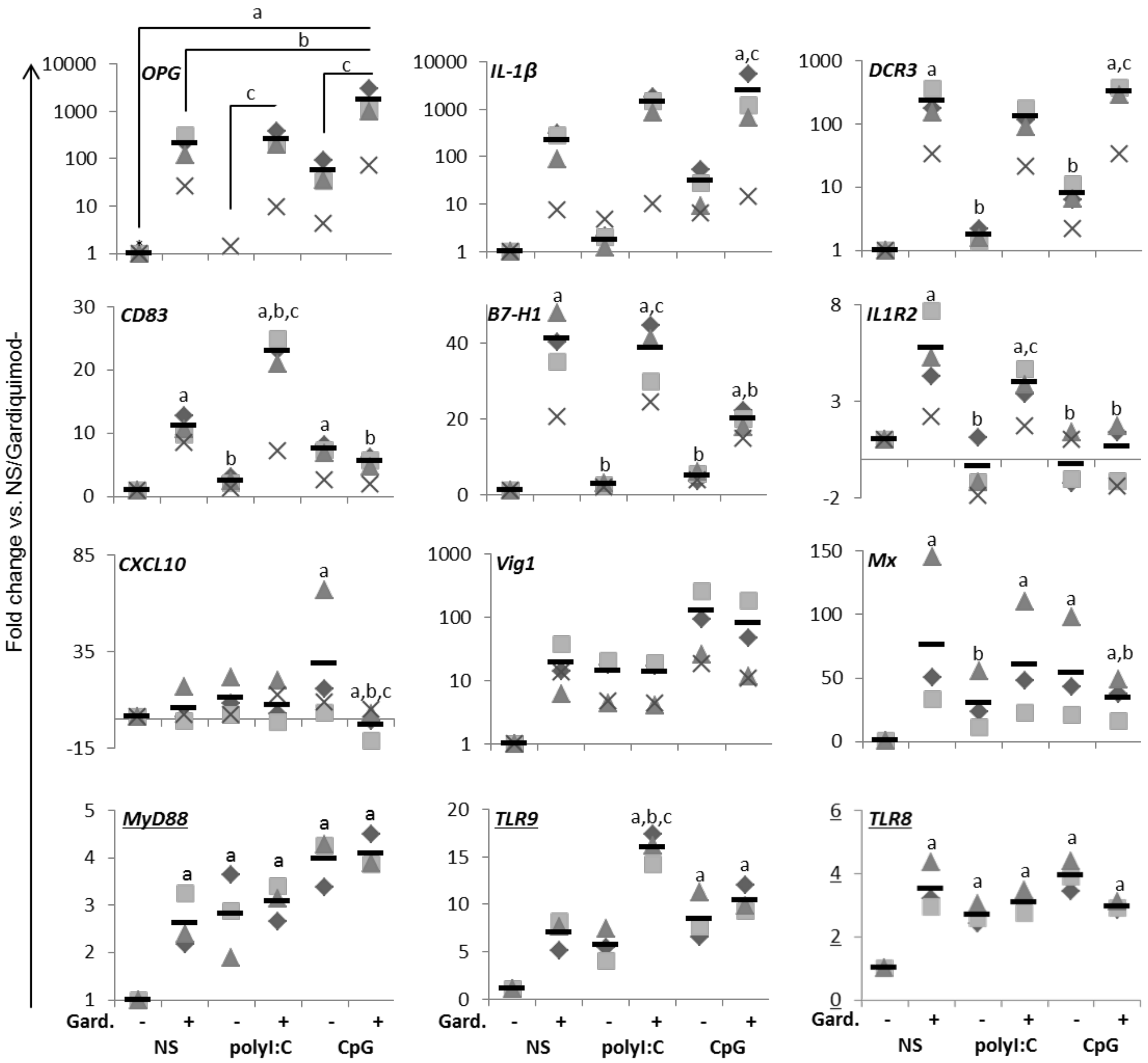


**Figure S2**. Real-time PCR analysis of gene expression in salmon MPs. RNA samples from three individuals (including the samples from the two individuals that were pooled for the microarray analysis) were analyzed separately. Samples restimulated with gardiquimod (gard.) are indicated with "+" below the X-axis. *EF1aB* expression was used as an endogenous control and the normalized RNA levels are presented as "fold difference" values as compared to the NS/Gard.- samples. The reaction conditions and data analysis were previously described (Front Immunol 2013. 4: 137). The data for each individual are represented with squares, triangles and diamonds. The mean values are shown with horizontal bars. Microarray data (if available) is shown with "X" . Note that for Mx, MyD88, TLR8 and TLR9, microarray data is not available. The data were analyzed with two-way ANOVA followed by Tukey's post test (a – p<0.05 against NS/Gard.-; b – p<0.05 against NS/Gard.+; c – p<0.05 against polyI:C/Gard.- and CpG/Gard.-).

**Table S1**. Sequences of the primers used in the PCR analysis.

| | | | |
|---|---|---|---|
| *B7-H1* (*GB accession#: NM_001141351)* | SYBR Green | Fwd | ACATGTGTCCAGGCTGAGGATCAA |
| | | Rev | ATTGTGGCAAGAGGATAGCCCTCA |
| *TNF decoy receptor 3 (DCR3)* *(GB accession#: XM_014146103)* | SYBR Green | Fwd | AGCATTGCACAAAGGACCGCAA |
| | | Rev | ACACCTTCTGCGCCTTGAACAT |
| *TNFRSF11B (OPG)* *(GB accession#: XM_014189272)* | SYBR Green | Fwd | ACGGGCCAGTTACTCACCTGTAAT |
| | | Rev | TGAGAACCGAGCATTCCTCCTTGA |
| *IL1R2* *(GB accession#: NM_001145420)* | SYBR Green | Fwd | AGCGAGATCACTTGGGAGGTGTTT |
| | | Rev | AAGTGTGTCACTCGAAACCAGGGA |
| *Vig-1* *(GB accession#: NM_001140939)* | SYBR Green | Fwd | AGTGCAGTTAAACAGGCGGA |
| | | Rev | ATATGCGTTTCCTGGATTGCCG |
| *CD83* *(GB accession#: XM_014200893)* | Taqman | Fwd | GTGGCGGCATTGCTGATATT |
| | | Rev | CTTGTGGATACTTCTTACTCCTTTGCA |
| | | Probe | CACCATCAGCTATGTCATCC |
| *IL-1β1* *(GB accession#: NM_001123582)* | Taqman | Fwd | GCTGGAGAGTGCTGTGGAAGA |
| | | Rev | TGCTTCCCTCCTGCTCGTAG |
| | | Probe | TTGGAGTTGGAGTCGGCGCCC |
| *EF1aB* *(GB accession#: XM_014141923)* | Taqman | Fwd | TGCCCCTCCAGGATGTCTAC |
| | | Rev | CACGGCCCACAGGTACTG |
| | | Probe | AAATCGGCGGTATTGG |
| *CXCL10* *(GB accession#: EF619047)* | Taqman | Fwd | AGGAGTGTGCAGTAAATCTGTGAAC |
| | | Rev | CTCATGGTGCTCTCTGTTCCA |
| | | Probe | CAATTCCACTAAGAACTTG |
| *Mx1/2* *(GB accession#: XM_014133087)* | Taqman | Fwd | GATGCTGCACCTCAAGTCCTATTA |
| | | Rev | CGGATCACCATGGGAATCTGA |
| | | Probe | CAGGATATCCAGTCAACGTT |
| *TLR8* *(GB accession#: NM_001161693)* | Taqman | Fwd | ACCAAAACCACTAATGACATCATCTTCA |
| | | Rev | TGGTGATGCCATCAGGTATGTTT |

| | | | |
|---|---|---|---|
| | | Probe | CTCAGTCGACGCTCCTC |
| *TLR9* *(XM_014134611)* | Taqman | Fwd | TCTATGGCTGGGATGTCTGGTA |
| | | Rev | CAGTTGTGAGTAGCCCTTGTGT |
| | | Probe | CAGCACCTGGAAGCAG |
| *MyD88* *(NM_001136545)* | Taqman | Fwd | GACAAAGTTTGCCCTCAGTCTCT |
| | | Rev | CCGTCAGGAACCTCAGGATACT |
| | | Probe | CTGGTGCCCGGAGCAA |
